# From design to action: participatory approach to capacity building needs for local overdose response plans

**DOI:** 10.1186/s12889-023-15414-3

**Published:** 2023-04-27

**Authors:** Maryam Mallakin, Christina Dery, Yordanos Woldemariam, Michael Hamilton, Kim Corace, Bernie Pauly, Triti Khorasheh, Caroline Bennett AbuAyyash, Pamela Leece, Katherine Sellen

**Affiliations:** 1 Health Design Studio, OCAD University, Toronto, ON M5T 1W1 Canada; 2 Institute for Safe Medication Practices Canada (ISMP Canada), Toronto, ON M2N6K8 Canada; 3 Royal Ottawa Mental Health Center, Ottawa, ON K1Z7K4 Canada; 4 University of Ottawa, Ottawa, ON K1N6N5 Canada; 5 University of Victoria, Victoria, BC V8P5C2 Canada; 6 Canadian Institute for Substance Use Research, Vancouver, BC V6Z2A9 Canada; 7 Health Promotion, Chronic Disease and Injury Prevention, Public Health Ontario, Toronto, ON M5G1V2 Canada; 8 Dalla Lana School of Public Health, University of Toronto, Toronto, ON M5T3M7 Canada; 9 Department of Family and Community Medicine, University of Toronto, Toronto, ON M5G1V7 Canada

**Keywords:** Opioid crisis, Overdose, Capacity-building, Public health, Participatory design, Co-design workshop

## Abstract

**Background:**

In response to the rise in opioid-related deaths, communities across Ontario have developed opioid or overdose response plans to address issues at the local level. Public Health Ontario (PHO) leads the Community Opioid / Overdose Capacity Building (COM-CAP) project, which aims to reduce overdose-related harms at the community level by working with communities to identify, develop, and evaluate capacity building supports for local needs around overdose planning. The ‘From Design to Action’ co-design workshop used a participatory design approach to engage communities in identifying the requirements for capacity building support.

**Methods:**

A participatory approach (co-design) provided opportunity for collaborative discussion around capacity building needs at the community level. The co-design workshop included three structured collaborative activities to 1) prioritize scenarios that illustrated various challenges associated with community overdose response planning, 2) prioritize the challenges within each scenario and 3) prioritize the supports to address each of these challenges. It was conducted with fifty-two participants involved in opioid/overdose-related response plans in Ontario. Participatory materials were informed by the results of a situational assessment (SA) data gathering process, including survey, interview, and focus group data. A voting system, including dot stickers and discussion notes, was applied to identify priority supports and delivery mechanisms.

**Results:**

At the workshop, key challenges and top-priority supports were identified, for development and implementation. The prioritized challenges were organized into five categories of capacity building supports addressing: 1) stigma & equity; 2) trust-based relationships, consensus building & on-going communication; 3) knowledge development & on-going access to information and data; 4) tailored strategies and plan adaptation to changing structures and local context; and 5) structural enablers and responsive governance.

**Conclusion:**

Using a participatory approach, the workshop provided an opportunity for sharing, generating, and mobilizing knowledge to address research-practice gaps at the community level for opioid response planning. The application of health design methods such as the ‘From Design to Action’ co-design workshop supports teams to gain a deeper understanding of needs for capacity building as well as illustrating the application of participatory approaches in identifying capacity building needs for complex public health issues such as the overdose crisis.

**Supplementary Information:**

The online version contains supplementary material available at 10.1186/s12889-023-15414-3.

## Background

Rising drug-related deaths is a global issue with a significant impact on population health and well-being. In 2016, Health Canada identified opioid–related harms as a national public health crisis with devastating consequences for individuals and families across the country [1]. In response, the Government of Canada’s first approach was to commit to taking evidence-based opioid response action through public health approaches including the four-pillars of the Canadian Drugs and Substance Strategy (CDSS): Prevention, Harm Reduction, Treatment, and Enforcement [2].

Although the overdose crisis has affected every province, British Columbia and Alberta have experienced the highest rates of opioid toxicity deaths in the country since 2016. The number of fatal and non-fatal opioid/overdose incidents has risen dramatically in Ontario, such that the province had the highest number of apparent opioid-related deaths in 2020 (2,430 deaths) [3]. These tragic numbers underscore the need for ongoing surveillance and comprehensive, multifaceted public health approaches to reduce and prevent overdose-related harms [4]. While opioid-related harms were an initial focus, a broader understanding of drug overdose recognizes that multiple substances are often involved, and as such we generally use the term overdose herein.

Many communities across Canada have developed multi-sector, multi-strategy overdose response plans to address the issue at the local level. Study of current community overdose response plans reveals gaps which need further research including: 1) the role and involvement of marginalized groups (e.g., people with lived/living expertise (PWLE), LGBTQ2SIA community, Indigenous and racialized communities), 2) the role of context (e.g., geographic, sociocultural, economic and political context), and 3) evaluation to understand the implementation of community opioid/overdose response plans [5].

Health Canada has funded Public Health Ontario (PHO) for a four-year project to develop a capacity-building model to support comprehensive community overdose response plans in Ontario employing a participatory approach. Co-design in health has played an increasingly important role in engaging communities in health system change and service delivery planning. The participatory processes and techniques support community-based decision making and shared ownership [6, 7], by including:

- co-located and contextually located engagement [8] as a necessary mechanism for uncovering community-based knowledge and factors that might impact successful implementation [9] of change,
- the use of physical manifestations of shared knowledge and understanding [10] in the form of design artifacts and prototypes [11], and,
- the use of structured dialogue and facilitation to support shared decision making and consensus building over time [12] towards solutions that are collectively shaped and owned [13].

These aspects of co-design are intended to support a process of more equitable change by engaging with multiple stakeholders and deliberately including the voice of those with lived experience in decision making, ownership, and the process of innovation [9]. As such this approach is particularly suited to the challenge of developing support for capacity building at the local level.

The Community Opioid /Overdose Capacity Building (COM-CAP) project is a collaboration between various sectors involved in community overdose response plans. The project aims to form partnerships across varied stakeholders in health units, drug strategy agencies, academia, and other sectors to develop, implement and evaluate a community overdose capacity building model to support comprehensive community opioid overdose response plans in Ontario.

Capacity building in this context refers to the ability of individuals, organizations, and also society as a whole to become able to define the issues at the local context by analyzing their environment, collaborating on managing and resolving conflicts, formulate strategies and provide action plans, and also to acquire and mobilize resources [14].

The project consists of four phases: a situational assessment, identification and design of the COM-CAP model and tools, implementation, and evaluation of the model. Overall, the project aims to reduce opioid-related harms at the community level through the following objectives 1) adapting a multi-component, evidence-informed, community development model; 2) examining, implementing and evaluating the proposed COM-CAP model with ongoing facilitation, knowledge brokering, and implementation supports; and 3) using integrated and knowledge translation processes to ensure the sustainability and scale-up of the model.

A co-design workshop (entitled “From Design to Action”) was conducted to provide an opportunity for collaborative discussion around capacity building needs and potential support at the local level as part of the phase two—identification and design of the project model and tools. The workshop used a participatory process with the intention that all diverse and relevant stakeholders represented on community overdose response plans should be involved throughout the project planning process, design, and development of the project tool (capacity building supports). This paper presents the results of the main breakout session 1 ‘From Design to Action’ co-design workshop. A second breakout session focused on how to evaluate COM-CAP’s anticipated impacts. The session began by presenting the “Framework for Evaluation of Complex Drug Strategies”, [19] followed by a facilitated group discussion activity. The results of breakout session 2 will be presented elsewhere.

## Methods

The project employed a co-design participatory approach for a deeper understanding of overdose capacity building needs at the local level and achieving more in-depth information and insight for developing the COM-CAP project tools. This approach also facilitated and supported inclusion of lived/living expertise, community-based decision making, consensus building, and shared ownership in the building capacity at the local level [6, 7]. The workshop techniques and materials (see Appendix A) were developed based on results achieved from a prior situational assessment which is reported in detail elsewhere [15]. In summary, the situational assessment identified four main themes in order: a) data and information; b) evidence and practice; c) implementation factors; and d) partnership, engagement and collaboration. Stigma and equity were noted as overarching areas of need to be addressed across all main themes. The elicitation techniques used to structure the workshop were intended to engage the participants in a collaborative discussion and identification of the issues experienced in leading community overdose response plans. Ethics review for the project was provided [removed for review] and [removed for review] Research Ethics Board. Informed consent was obtained from all participants.

Workshop participants included 52 representatives from public health, academic, government, and community sectors involved in opioid and overdose-related plans in Ontario. Participants included representatives of public health units and drug strategies in Ontario (*n* = 22), hospitals (*n* = 4), provincial government or agencies (*n* = 3), academia (*n* = 2), 17 participants from other involved sectors in opioid-related plans including harm reduction efforts, and 10 people with lived/living expertise of drug use.

Co-design practices are often intended to deal with power differentials between stakeholders, which if not addressed appropriately can impact co-design’s effectiveness [16]. Having skillful facilitators, creating an inviting space for participants to easily engage and interact, and shifting power from people to the process, are strategies that can mitigate power imbalance in the co-design process [17] To mitigate potential issues of positionality and power differentials in the workshop and provide participants the opportunity to engage in the discussion and shift the power to the process, the following techniques and tools were used to provide better opportunities for participants to engage and express themselves: the 52 participants were invited to self-organize into 12 groups of 5/6 with 1 facilitator per group – facilitating choice and agency, facilitators were all trained in supportive practices and regularly involved in substance use projects, investigators/project leads/ budget holders were not involved in facilitation or participation, scenarios and personas (as subjects of conversation), prompt cards (as tools for conversation), and a community capacity building matrix (as a central enabler in generating and forming new ideas on priority supports) were provided as a scaffold for activity focused dialogue directed to community based needs [18]. Scenarios were the main mechanism used for encouraging communication and collaborative discussion. Personas and scenarios used in a healthcare context enable participants to engage more quickly in topics during participatory workshops since they enable speaking to and through a fictional scenario and character. Personas and scenarios serve as a skeleton framework, enable participants to relate with the specific situation and character, flesh out details from their perspective and discuss the needs and high-level actions for addressing the needs [19]. Personas are a vivid, fictitious representation of a specific character, which has a potential to build and develop empathy with the real character. It enables discussion about a rich and authentic personality with specific needs [20].

The technique of using personas and scenarios is also effective in situations where participants may not have continuous involvement in a change process. It allows the specific needs of participants to be accounted for, ensuring fuller engagement with those who may usually not be invited to contribute and provide input [21].

**Fig. 1:**
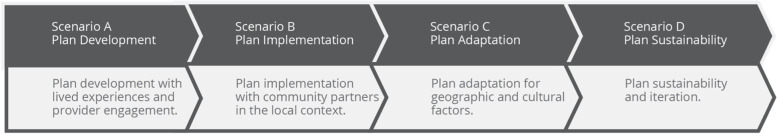
Workshop Scenarios

Four workshop scenarios were developed from themes that emerged from the situational assessment process. The scenarios were designed to illustrate situations that embodied the themes across different phases of the community overdose response planning, including Plan development, Plan implementation, Plan adaptation, and Plan sustainability & iteration (Fig. 1). (For more details of the scenarios see Appendix A, supplementary Figs. 1–4).

Various prompt cards were developed as tools for conversation (see examples in Appendix A, supplementary Figs. 6–8). We used persona cards (see example in Appendix A, supplementary Fig. 5) as a realistic representation of diverse stakeholders involved in opioid/overdose plans, including: local paramedic, local pharmacist, shelter director, drug strategy coordinator, family physicians, PWLE; quote cards and wild cards were developed to represent specific types of ideas and situations, and 40 challenge cards were developed and designed to address the most important identified areas where support was needed for the key themes across the workshop scenarios.

Capacity building matrix was developed to enable discussion and ideation on more practical aspects of how challenges could be supported through capacity building ideas. The matrix provided support at the individual and organizational levels (see Appendix A, supplementary Fig. 9).

**Fig. 2:**
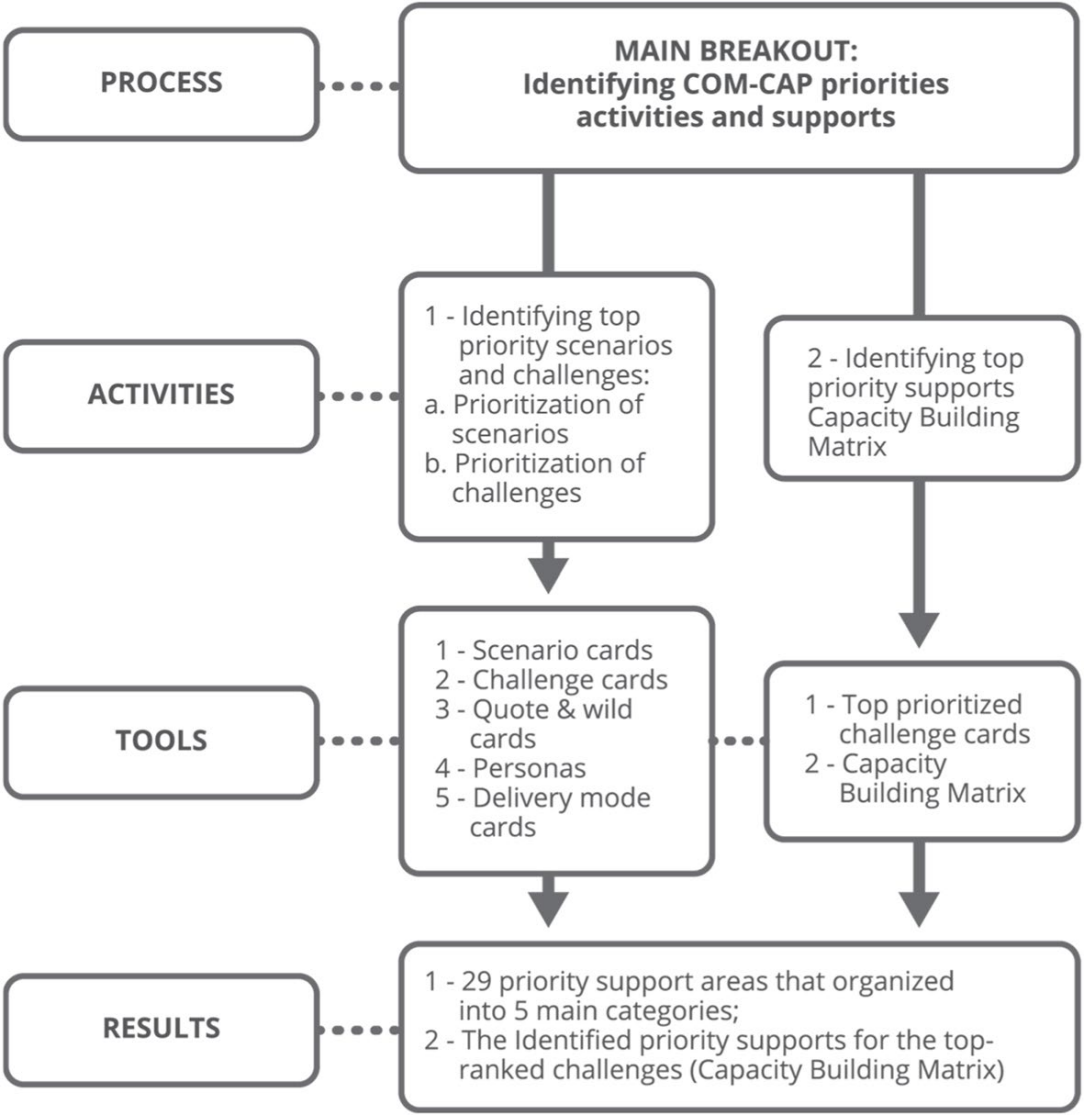
Co-design workshop process

The workshop consisted of a breakout session, which aimed to provide a space to collectively discuss and identify the top-priority COM-CAP scenarios, challenges and supports. Figure 2 illustrates the workshop process.

### *Breakout session:* COM-CAP top-priority scenarios, challenges, and supports

The breakout session identifying the top-priority COM-CAP scenarios, challenges and supports consisted of two activities (Fig. 2):

1. Identifying Top-Priority Scenarios and Challenges
  - Step 1: Prioritization of Scenarios (selection of two scenarios to focus and work on)
  - Step 2: Prioritization of Challenges for the selected scenarios
2. Identifying Top-Priority Supports—Capacity Building Matrix

### Activity one: Identifying top priority scenarios and challenges

Participants were divided into 12 multi-stakeholder groups for facilitated co-design activities. Each group consisted of 5 participants and a facilitator to introduce workshop materials and facilitate collaborative discussion on co-design activities. Each group was provided with four scenarios, and associated challenge cards, personas, quote cards, and wild cards. Participants were asked to:

- Review and prioritize each scenario and choose two to work on;
- Record their rationale for scenario choices;
- Review, select, and prioritize the top challenges for each scenario (participants had the opportunity to add other challenges that were not already represented).

### Activity two: Identifying top priority supports

To delve deeper into the most urgent or priority supports for the COM-CAP project, each group was asked to:

- Collaboratively identify supports/resources/tools that would enable capacity building for top priority needs;
- Use a capacity building matrix to structure a discussion and ideation session considering both individual and organizational level needs under five specific topics: Support for whom? to do what? how to develop? deliver? and sustain?
- Vote on the top three challenge cards and priority supports (using dot stickers on the matrix).

Figure 2, illustrates the process for breakout session 1 (Identifying COM-CAP top-priority scenarios, challenges and supports), including: the session process, employed activities, applied tools, and achieved results.

### Analysis

Discussion notes and the 19 capacity building matrices completed by participants (short text-based contributions on sticky notes), were collected, maintaining participants’ prioritization of the key areas where support was needed (challenges) and detailed components of priority supports. We analyzed participant contributions using a deductive qualitative approach for text-based contributions [23, 24] due to the a priori structuring of the co-design materials and prompts. Analysis was undertaken by the research team at OCAD University including 4 research assistants and 1 senior researcher, this process included the transcription of written notes and sticky notes into Microsoft Excel. The first step was to maintain the categories by scenario, challenge and support matrix in line with the structuring of the co-design session. These initial categories were then reviewed by the team, revising the sorting of data within each five major categories as discussion developed around convergent and divergent participant contributions (within a priori structure of the data). This process was undertaken three times before developing descriptions of for priority challenge areas, and collaboratively describing each support that could address these challenge areas. Participants’ votes enabled the research team to rank the challenge areas and the supports to address these challenges. The associated delivery methods, that were to be considered in the design and development of the main components of the COM-CAP project tool were maintained through this process. The priority challenge areas where support was needed were then shared with the advisory and scientific team, and then the community collaborators for feedback and review. No adjustments to the priority challenge areas were made at that stage.

## Results

An overview of the main findings from co-design activities are discussed below.

Key findings: Identifying COM-CAP top-priority scenarios, challenge areas and supports

### Activity 1: Identifying top-priority scenarios and challenge areas

#### Step 1: Identifying top-priority scenarios

The first activity began with each group prioritizing 2 top scenarios and recording the rationale for those choices. The results indicate that two scenarios, B followed by A, were consistently prioritized for further work across the groups, indicating a focus on capacity-building supports that address partnerships with community partners and providers (Scenario B: Plan Implementation with Community Partners in the Local Context; Scenario A: Plan Development with Lived experiences and Provider Engagement). Scenario C was considered to be more specialized and viewed as a second step after Scenario B and A were underway (Scenario C: Plan Adaptation for Geographic and Cultural Factors). Scenario D was not prioritized for work by any of the groups; however, four groups were invited to work on Scenario D so that information about challenges related to it would be represented as the project moved forward (Scenario D: Plan Sustainability and Iteration).

#### Step 2: Identifying top priority challenges

**Fig. 3:**
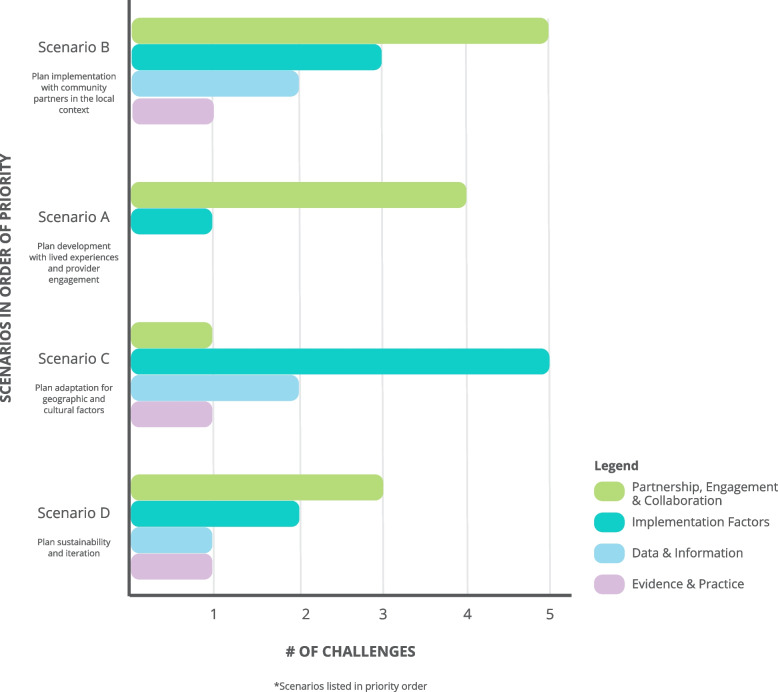
Identifying top priority scenarios and challenges (colors indicate the theme areas identified during the situational assessment)

Each group was responsible for identifying and prioritizing areas where support was needed (challenges) for each selected scenario. Challenges related to partnership, engagement and collaboration, as well as implementation were the main areas of focus that emerged from the co-design workshop. The number of challenges identified within each scenario are color coded based on the four themes from the SA and can be seen in Fig. 3.

**Fig. 4:**
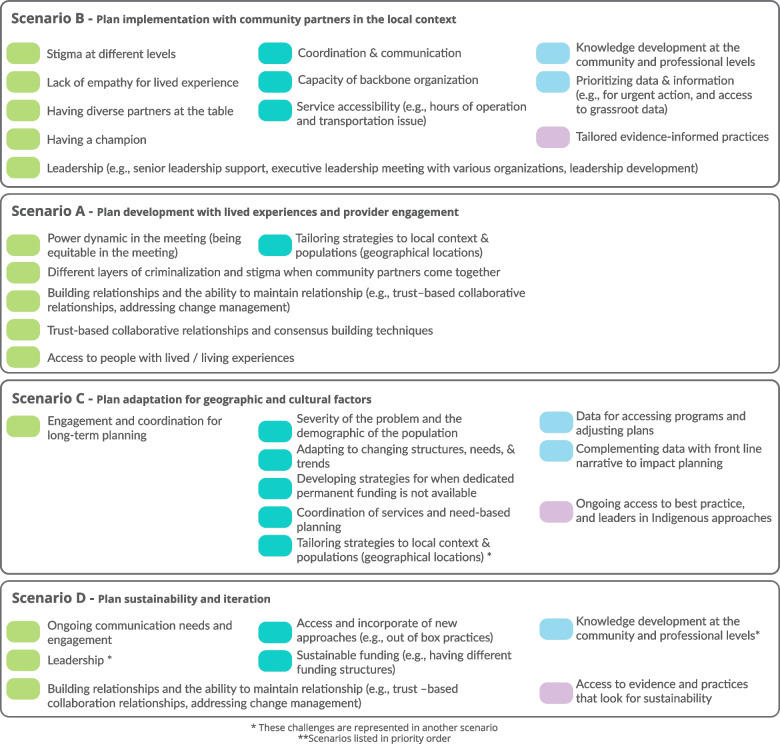
Identifying top priority scenarios and challenges (colors indicate the theme areas identified during the situational assessment)

A total of 29 challenge cards were selected/created overall (some new challenges were identified by participants on blank cards). Appendix B, supplementary tables 1–4 present the detailed results of this activity. The results of the first activity were organized across the four main scenarios and color-coded based on the main SA themes by the research team. Fig 4 below shows the prioritized challenges for each scenario, as determined by the participants.

**Fig. 5:**
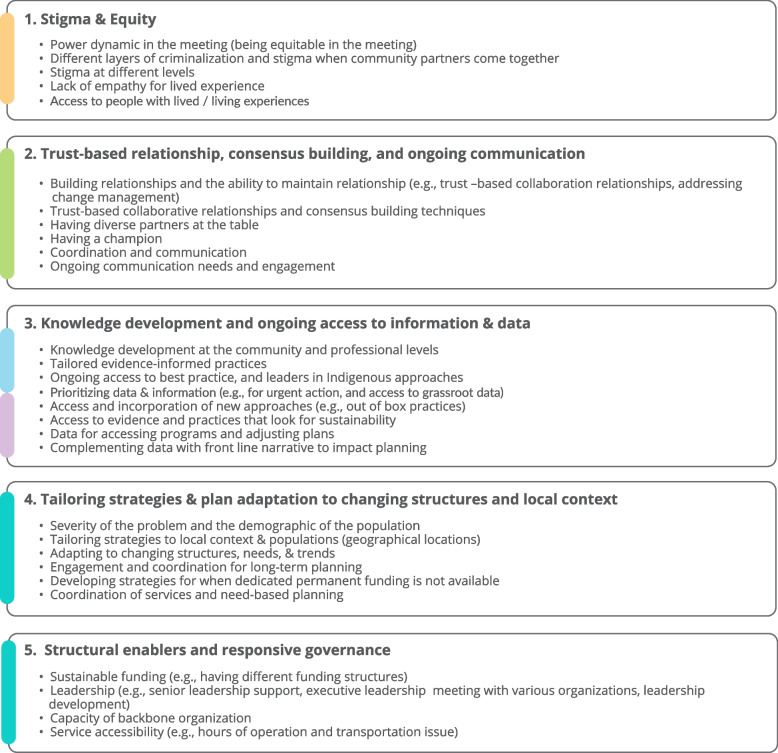
Prioritized challenges organized into five major categories (colors indicate the theme areas identified during the situational assessment with the addition of the overarching theme of stigma and equity in golden yellow)

The research team grouped these 29 priority challenges into five main categories through thematic analysis. Fig 5 shows the five main identified categories and all their challenges.

### Activity two: Identifying top priority supports

**Fig. 6:**
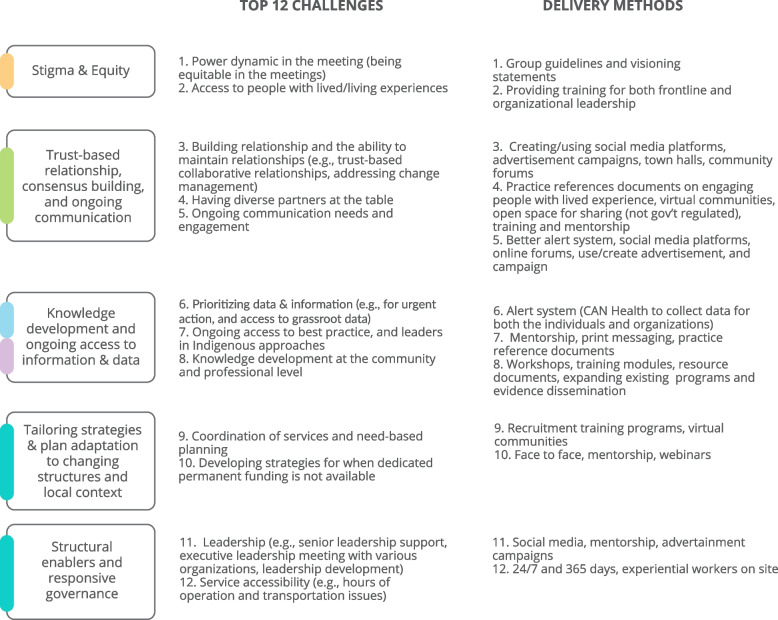
Top 12 challenges across categories with corresponding supports and delivery methods as identified by workshop participants

Further to the development of the five categories above (Fig 5), the 3 top-ranked challenges were selected for each scenario supported by participants’ votes. Fig 6 below shows the top 3 ranked challenges for each category.

Following the first activity, each group was responsible to discuss and identify priority supports to address the 3 prioritized challenges, including corresponding methods to deliver the supports. The identified priority supports for the 3 top-ranked challenges are presented in Appendix C, supplementary tables 55–16.

The table below mechanisms for delivering supports alongside the top 3 challenges by category.

The delivery methods were grouped into three main categories, to be considered in development of the project tool(s), including:

1. Resources (online/in-person) for maintaining healthy and active communities (as an essential element to support plan development and implementation)
2. Concrete training materials using different media
3. A suite of templates/tip sheets/guidelines for running meetings/decision making techniques

Development of an alert system and data dissemination methods was identified as an additional category but treated separately as it relates specifically to an alert system for contaminated drug supply at the local level.

### Priority supports

**Fig. 7:**
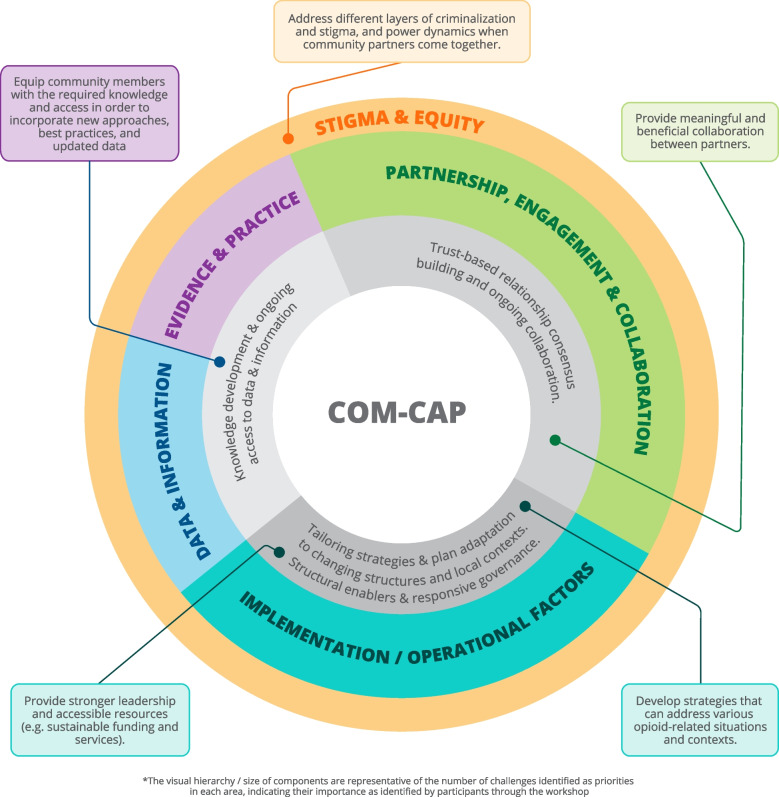
Visualization of project themes and major priority supports identified in the workshop

The model (Fig. 7) was developed based on the key findings of the COM-CAP co-design workshop. It presents the main areas to be considered and developed to address local needs around opioid and overdose plans.

The model is broken down into three main rings:

- Ring 1and 2: The categories identified from prioritized challenges in the workshop: 1) partnerships, engagement and collaboration, 2) implementation factors, 3) data and information, and 4) evidence and practices, segment size reflecting participant priorities. Stigma and equity as overarching category that impacts everything within the model.
- Ring 3 (inner ring in grey)):Three areas of prioritized challenges from the workshop which are conceptually similar and presentd here with example contributions from participants (callouts).

## Discussion

This paper highlights activities in the planning stage of our project, facilitated by co-design. The initial situational assessment provided an in-depth understanding that resulted in a source of data including challenge areas as they relate to community overdose response plans (strengths, gaps, needs, etc.) in Ontario. The second stage of adaptation and planning (co-design workshop) helped prioritize the challenges, which were organized into five major categories; potential supports to address each of the challenges were discussed in this process. The preliminary four categories of support from the situational assessment were refined through this process to develop the model in Fig 6 and Fig. 7. Through this process, detailed delivery methods and ideas for specific supports and tools were captured. These prioritizations and delivery methods will be instrumental in guiding the project in the next stage: identification and design of project tool(s) as this framework and information is used to develop capacity building support.

Our findings suggest that the community overdose response plans have several needs that require capacity building to support the development and implementation of plan objectives and goals. Capacity building is defined as the development of knowledge, skills, commitment, structures, and leadership to address challenges and improve health in three ways: the advancement of knowledge and skills, expansion of support, and development of engagement, partnership, and collaboration in communities [25]. Capacity building support can be delivered in various forms, including technical assistance, virtual and in-person training sessions, online learning options, and guidance materials (e.g., knowledge products). However, organizations should carefully consider the desired outcomes and select forms effective to those outcomes [25].

The identified and prioritized delivery methods in the workshop for building capacity included the use of online/in-person resources, social media platforms, a variety of training materials and adaptable guidelines. Addressing stigma and equity is another challenge that needs to be addressed when developing various educational programs and guidelines for providers and should therefore receive additional consideration. For example, understanding and addressing the experiences of PWLE [26] and emphasizing appropriate and non-stigmatizing language [27] can address opioid-related stigma among providers through educational programs and guidelines.

### Insights on Co-design process

A co-design methodology was used to guide the development of this project to support the participation of individuals with varied expertise in a manner that is engaging and easy to understand. The design process endeavored to share, mobilize, generate, and activate knowledge, specifically in complex systems such as community-based health care innovations [6–28].

As co-design methodology has been increasingly utilized in healthcare system approaches, more knowledge is needed on the factors that impact the effectiveness of these methods. The main influential factors in implementation of a co-design approach include: collaboration, practical and organizational factors, process and methods, and skills in facilitating and utilization of outcomes. The co-design approach plays an essential role in sharing and co-creation of knowledge, addressing power dynamics and positionality, negotiation of controversies, and generation of new ideas and solutions, which is a necessary mechanism for uncovering community-based knowledge and factors that might impact successful implementation of change [9–29].

### The workshop enablers and challenges

The evaluation of the COM-CAP co-design workshop showed some considerations around the enablers and challenges of the workshop. Enabling factors included: high levels of expertise, a great mix of participants to share vulnerable perspectives, the role of facilitators, productive group discussion, having frontline people and people with lived/living expertise of drug use at the table, and workshop materials (pre-made cards and matrix) that supported activity focused productive discussion.

We also identified two barriers to effective co-design, including:

- Participant fatigue as a challenge for solving problems at such a high level; components of the breakout sessions were found to be complex, difficult to understand and process; workshop activities were heavily layered and tasks were noted to be too intensive, specifically for PWLE;
- Facilitator observations that breakout groups had similar populations, geographic area and size; different group sizes; lack of equal representation of PWLE in all groups (due to COVID-19 many invitees were not able to attend the workshop); and time constraints for networking. They found that allocating more time for networking and informing participants in advance of the specific discussion topics and workshop activity details would have been beneficial.

The evaluation results of the COM-CAP workshop can help identify key barriers in co-design research processes to inform future practices. Insights gathered from this study could enhance the foundation and application of participatory design in the healthcare domain.

Previous research on strategic research partnerships with PWLE and peer organizations, highlights the importance of having meaningful participation of PWLE in research and policy through an environment that enables and values both leadership and contribution [30]. This includes providing visibility and recognition of these partnerships with peer organizations and groups in the broader research, government and health service sector [30]. The continued use and refinement of the co-design techniques used in this study could enhance the engagement of PWLE in the project's upcoming phases and in similar projects and initiatives. Blomkamp (2018) [31], notes that co-design within the public sector can often be used as a more effective, democratic and innovative alternative to community engagement, public participation and policy development. While barriers were observed in this project, co-design approaches can provide meaningful and visible participation of relevant and diverse stakeholders which can enhance cross-sectoral collaboration, the integration of local knowledge and experience, power-sharing at the individual and community-level and further support community engagement. Its important to note that co-design in public health related projects with marginalised groups has received critique and is an area of rapid development of new perspectives and practices [32]

The next stage of this work is two-fold, 1) the development of supports and capacity building tools aligned with the results of this workshop that are applicable across the sector and can be distributed widely, 2) the development of tools and supports with three community partner projects where participatory processes will guide the development of practical supports to meet localized needs with learnings that can be shared and adopted across the sector.

## Conclusion

We proposed a community opioid/overdose capacity building model consisting of more specific individual components based on key findings from the “From Design to Action” workshop. The capacity building model emphasizes the importance of partnerships, engagement and collaboration; knowledge development and ongoing access to data and information; leadership; and addressing stigma and equity in overdose response plans in the local communities.

In the following stages of the project, the key findings will be translated into project tool(s) to build capacity with local communities to support issues and needs experienced in community overdose response planning.

The selection of local community initiatives for support (pilot sites) will occur in the following phase, and these initiatives will be considered in the development process of the project tool(s), and include consideration of, including applicability of project tools when applied to different contexts and communities. This will ensure that differences in demographics, geography, culture, and other factors are accounted for and reflected in the feasibility and adaptability of project tool(s).

## Supplementary Information

**Additional file 1:** Appendix A

**Additional file 2:** Appendix B

**Additional file 3:** Appendix C

## Data Availability

The data gathered from the data gathering process and workshop will not be made publicly available. This is to ensure the privacy and confidentiality of our study participants is protected. However, datasets used and/or analysed during the current study are available from the corresponding author on reasonable request.

## Abbreviations

PHO: Public health ontario
COM-CAP: Community opioid / Overdose capacity building
SA: Situational assessment
CDSS: Canadian drugs and substance strategy
PWLE: People with lived/living expertise
