# Supplementary material for: From design to action: participatory approach to capacity building needs for local overdose response plans
Source: BMC Public Health. 2023 Apr 27;23:774. doi: 10.1186/s12889-023-15414-3 (PMC10132919; doi:10.1186/s12889-023-15414-3)
Supplement: Supplementary file 1 — Additional file 1: Appendix A [file 12889_2023_15414_MOESM1_ESM.docx]

**Appendix A**

**Workshop Activities & Materials**

The workshop activities and materials were developed based on the data and information that was achieved from the four main sources of the SA process including:

1. Document Review: 26 documents of community opioid plans across health units, drug strategies, LHIN, and other lead organizations in Ontario. The documents were included 23 community plans and 3 consultation reports that focused on opioid/overdose;
2. Key Informant Interviews: 10 interviews were completed, which included provincial/state staff that support community plans, evaluators, coordinators of community plans, and partner organizations;
3. Focus Groups: 3 focus groups were held, which included 25 people from public health staff, drug strategy coordinators, and other community leads across Ontario;
4. Online Survey: The online survey was sent to public health units’ staff and community drug strategy coordinators. Participants were asked to rank different types of supports based on importance and also, to develop a list of priorities COM-CAP should focus on.

A series of tools (materials) were developed and designed to facilitate discussion, including: scenario cards, persona cards, challenge cards, quote and wild cards, delivery mode cards, and capacity building matrix. Each of these tools/materials will be discussed further below.

**Scenarios**

Scenarios are real stories that bring concepts to life. These stories enable readers to empathize with the people in the situation, which leads to questions about experiences, motivations, actions, and needs. Scenarios are both concrete and flexible and enable rapid communication and collaborative discussion among different stakeholders through raising questions at different levels. The discussion goes beyond the people and situations that are written in a scenario, and addresses challenges at multiple levels which leads to an in-depth understanding of the topic (Rosson & Carroll, 2002).

The four main applied scenarios in the workshop have come from the PHO’s themes- which emerged out of the Situational Assessment process. Each scenario represented relevant situations and stages in development of the community opioid action plan process, these included: Plan Development, Plan Implementation, Plan Adaptation, and Plan sustainability & Iteration. Each scenario also corresponded to the identified project themes: Partnership, Engagement & Collaboration, Implementation Factors, Data & Information and Evidence & Practice.

*Scenario A: Plan Development with Lived experiences and Provider Engagement*


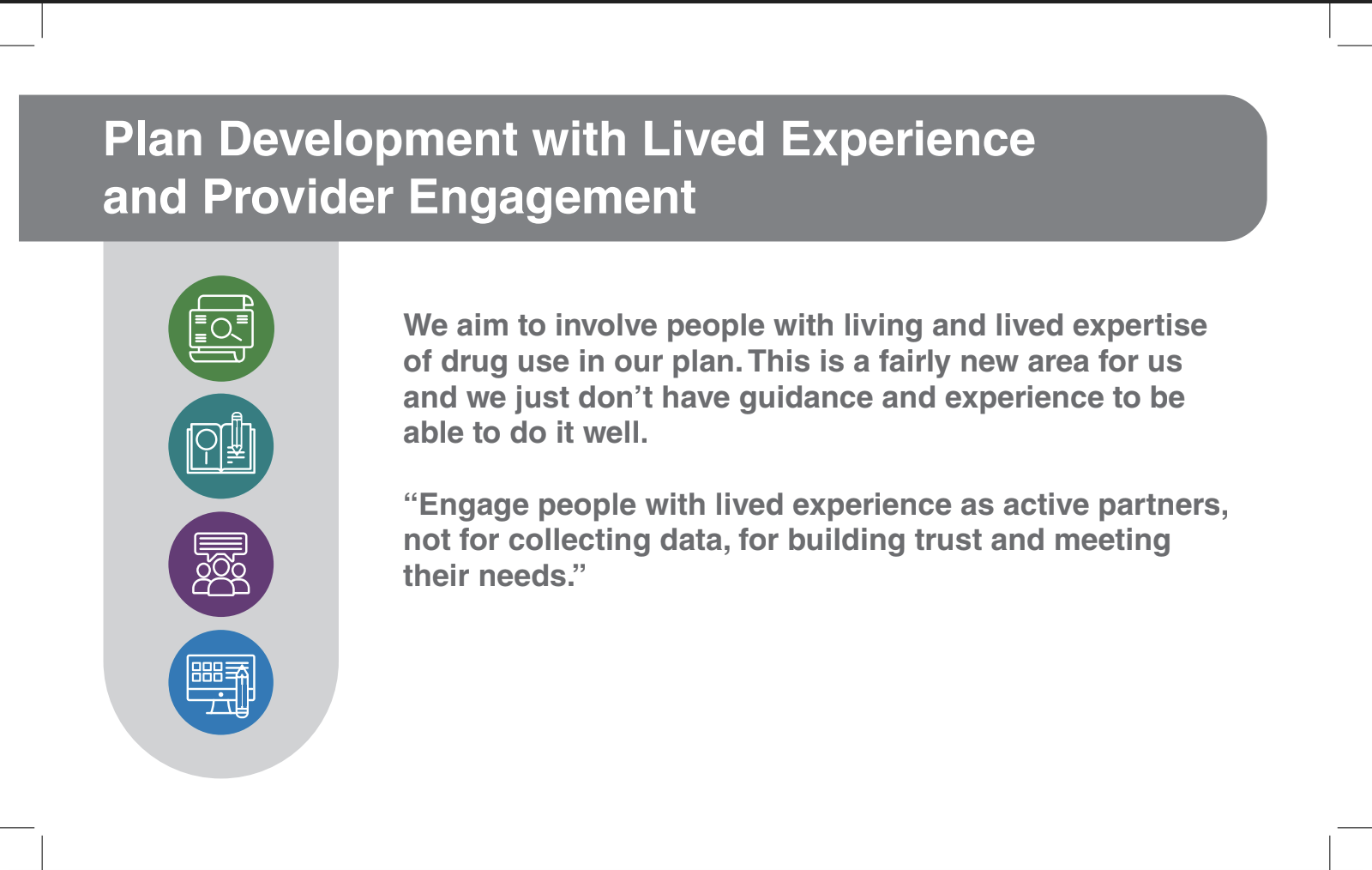


*Supplementary Figure 1: Scenario A workshop card. Coloured icons on the left represent the four different project themes: Partnership, Engagement & Collaboration, Implementation Factors, Data & Information and Evidence & Practice.*

Engaging people with lived/living experiences and providers in plan development is one of the challenges that is a fairly new area for some communities, especially when they don’t have guidance and experiences to be able to do it well. This scenario covered the four main themes and aimed to explore challenges and gaps in this area that need to be addressed.

*Scenario B: Plan Implementation with Community Partners in the Local Context*


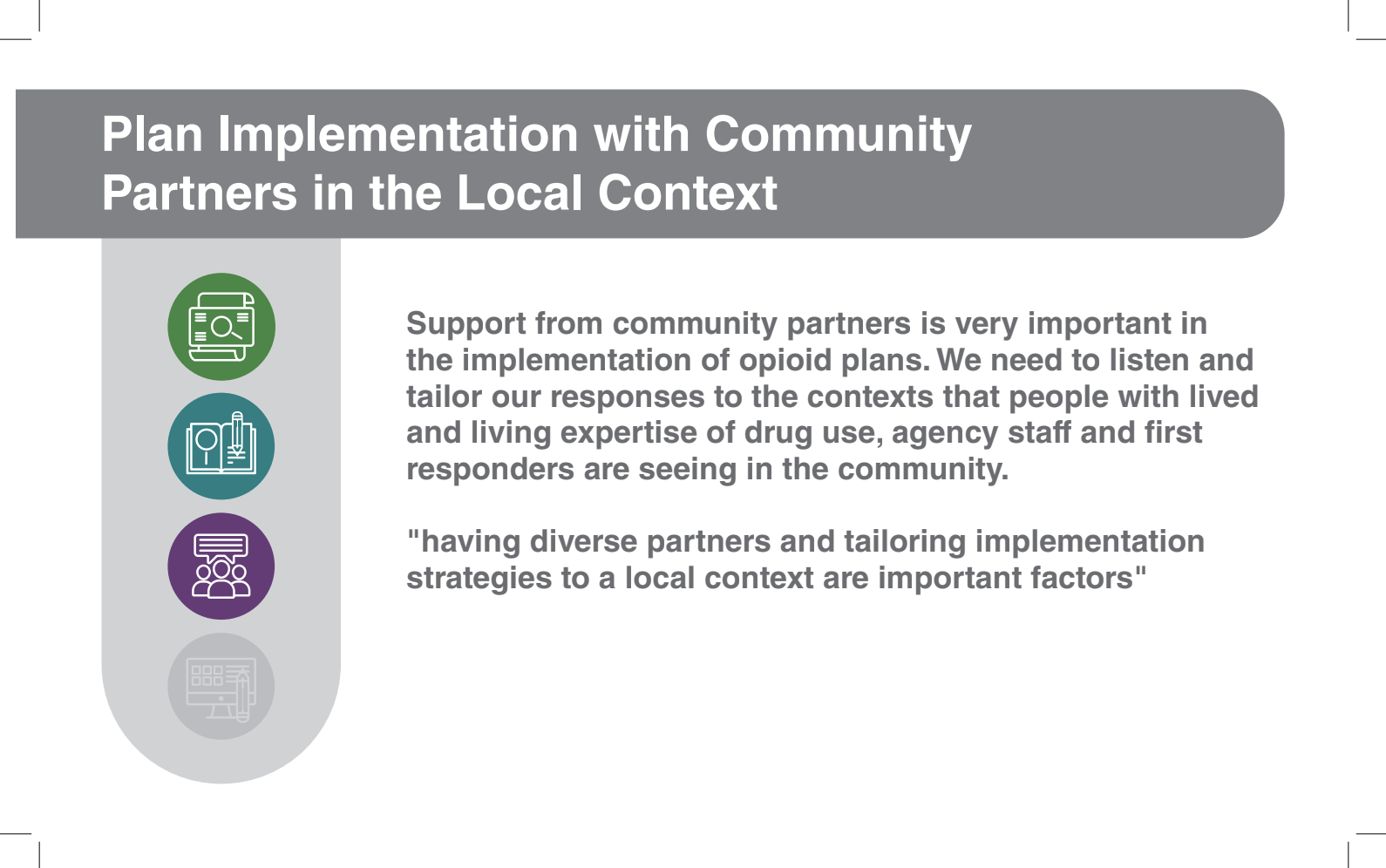


*Supplementary Figure 2: Scenario B workshop card. Coloured icons on the left represent three of the four different project themes: Partnership, Engagement & Collaboration, Implementation Factors and Evidence & Practice.*

Having diverse partners and tailoring implementation strategies to a local context are two main factors in implementation of the opioid plans. There are various challenges in bringing diverse partners at the table and tailoring implementation strategies to a local context. This scenario covered three themes (Evidence and Practice, Implementation Factors, and Partnership), and aimed to identify the most important challenges that affect implementation of opioid-related plans in the local context.

*Scenario C: Plan Adaptation for Geographic and Cultural Factor*


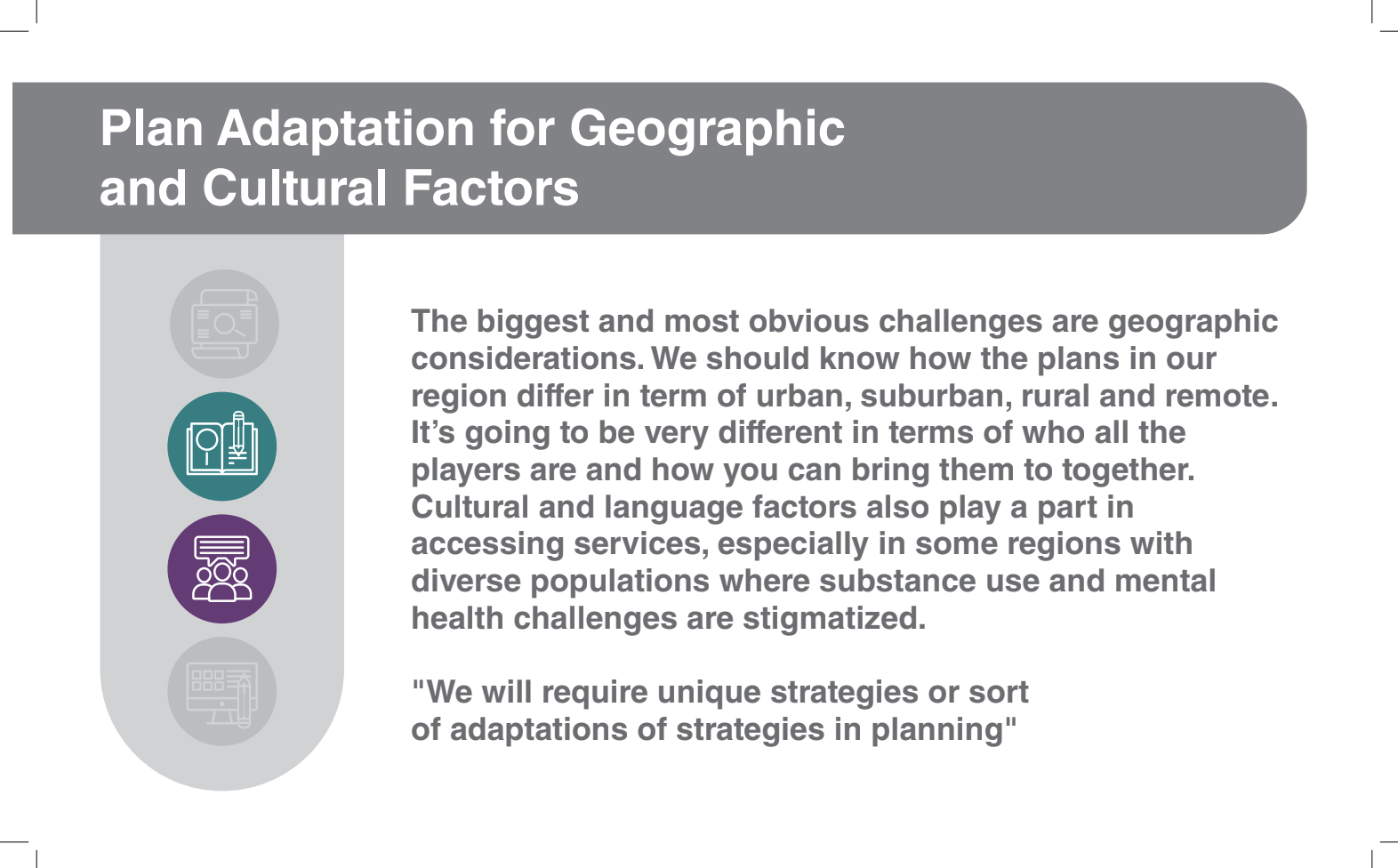


*Supplementary Figure 3: Scenario C workshop card. Coloured icons on the left represent two of the four different project themes: Partnership, Engagement & Collaboration & Implementation Factors.*

This scenario covered the ‘Implementation Factors’ and ‘Partnership’ themes and aimed to explore one of the biggest and most obvious challenges in plan adaptation that is the consideration of geographic and cultural factors.

*Scenario D: Plan Sustainability and Iteration*


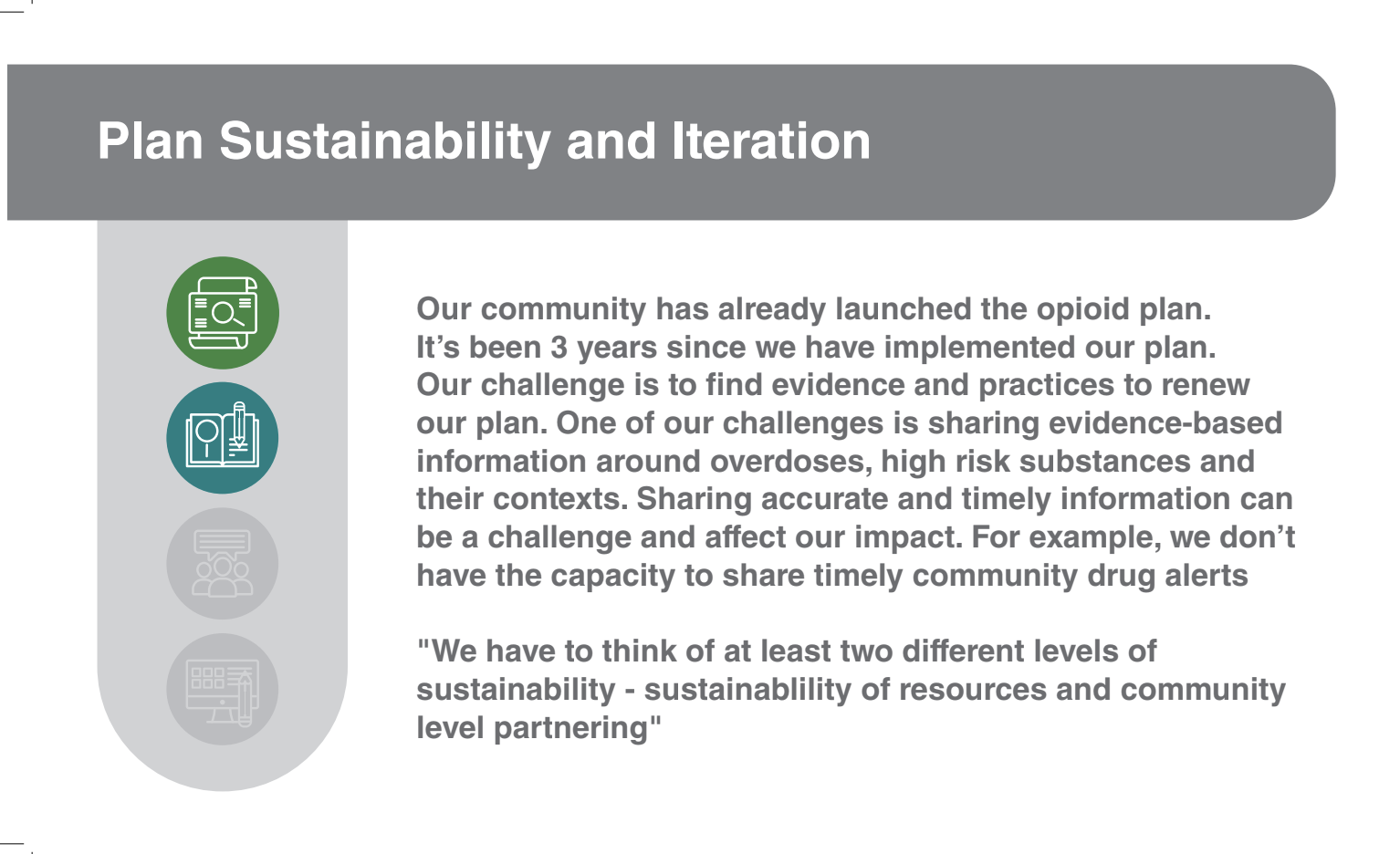


*Supplementary Figure 4: Scenario D workshop card. Coloured icons on the left represent two of the four different project themes: Implementation Factors and Evidence & Practice.*

Plan sustainability and iteration is one of the challenges of many communities that have already launched and implemented their opioid plan and are looking for evidence and practices to renew and support sustainability of the plan, specifically at two levels - sustainability of resources and community level partnering. The scenario D covered two themes (‘evidence and practices’ and implementation factors’) and aimed to identify the supportive strategies that address plan sustainability.

**Persona Templates**

Personas are realistic representations of diverse stakeholders. Personas develop from information gathered through research methods and help to visualize various experiences, motivations, actions, and needs. The workshop personas were developed from gathered information through the SA and aimed to communicate diverse core motivations and needs around the opioid-related plan within the communities.

**
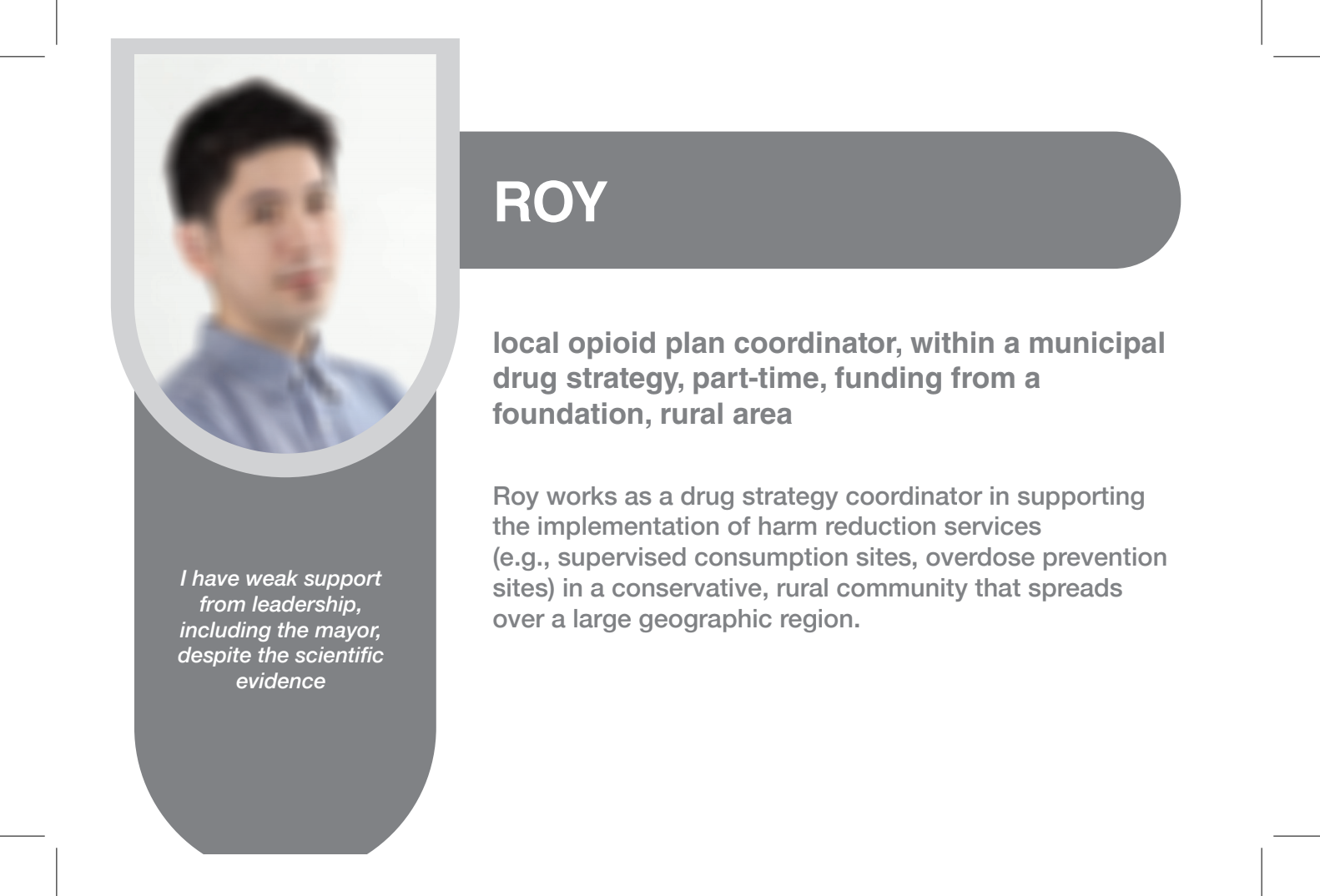
**

*Supplementary Figure 5: Example of a persona card used in the workshop.*

*Image source: https://www.dreamstime.com/stock-photo-serious-chinese-man-close-up-mature-image48491813*

**Challenge Cards**

Challenge cards: The 40 challenge cards were developed, designed, and grouped to four main sets (card deck). Each set addressed the most important identified support areas for the key themes across a specific phase of project planning, including: plan development, plan implementation, plan adaptation, and plan sustainability and iteration.


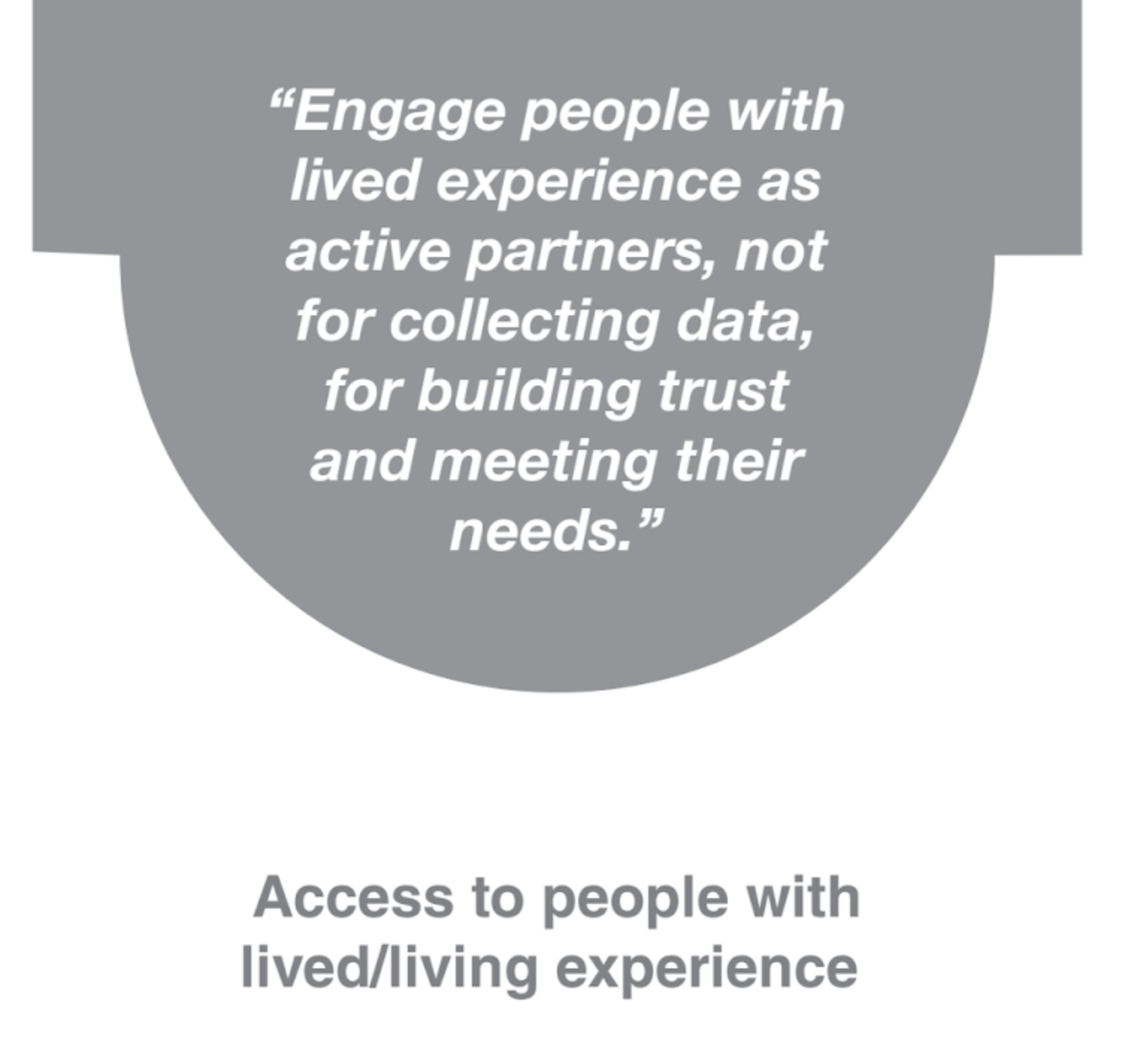


*Supplementary Figure 6: Example of a challenge card used in the workshop.*

**Quotes & wild Cards**

Quote and Wild cards: These cards were provided to bring more insights to the group discussion by representing specific types of ideas and situations.


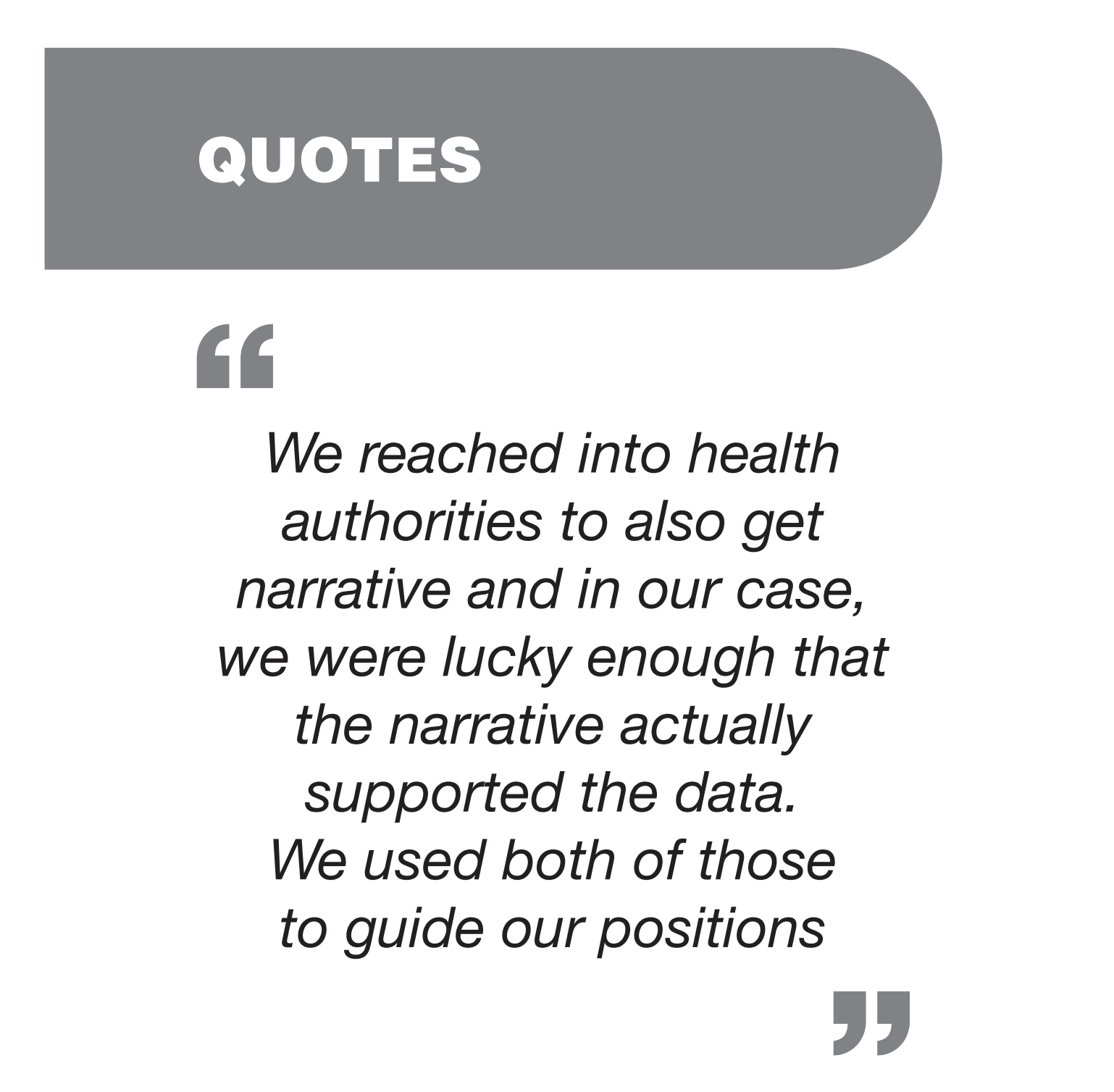

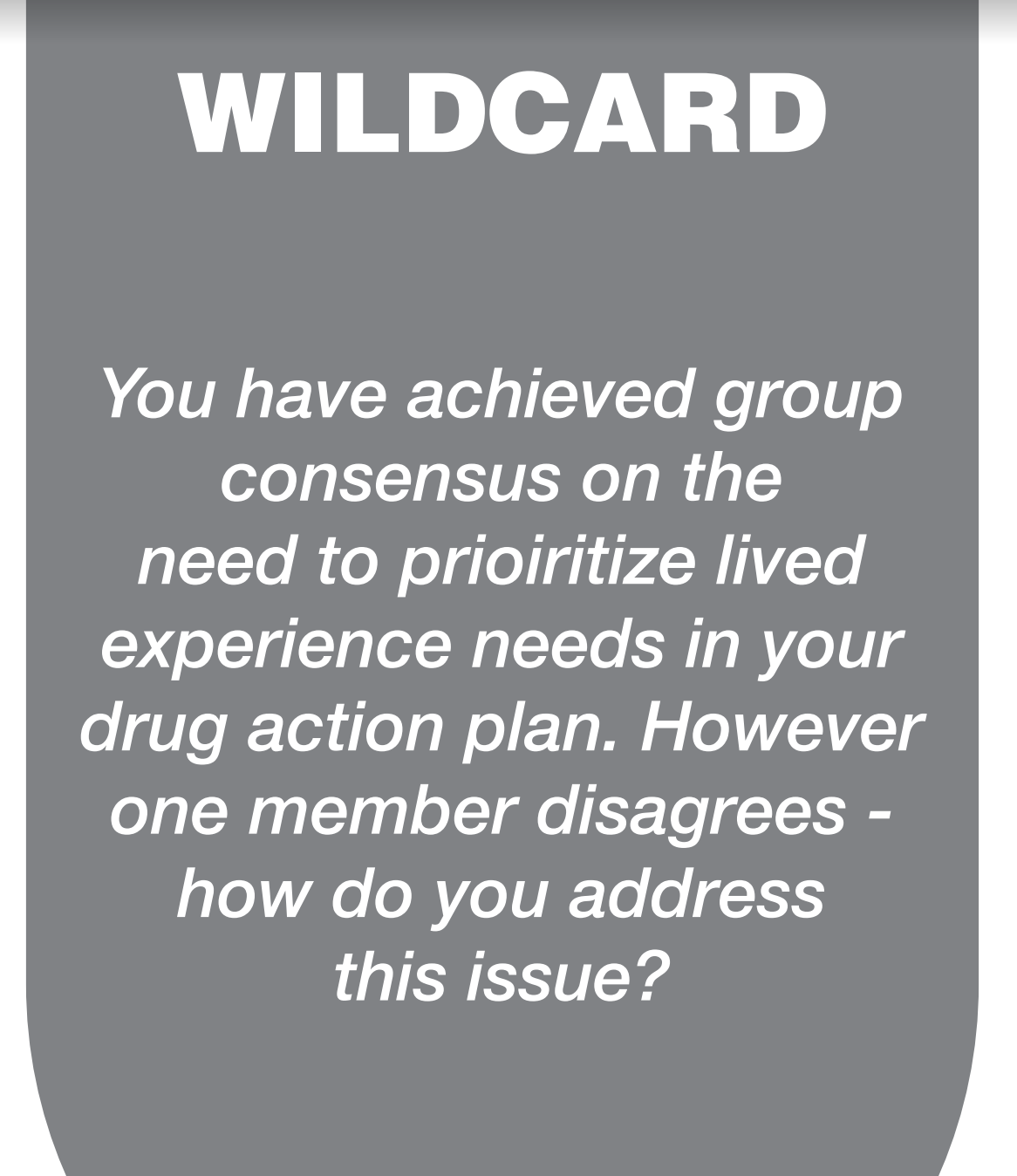


*Supplementary Figure 7: Examples of a quote card (left) and wild card (right) used in the workshop.*

**Deliver Mode Cards**

Delivery Mode cards: The delivery mode cards were created to provide an opportunity for the participants to explore how the recommended supports would influence the outcomes of the scenarios that they had chosen and prioritized.


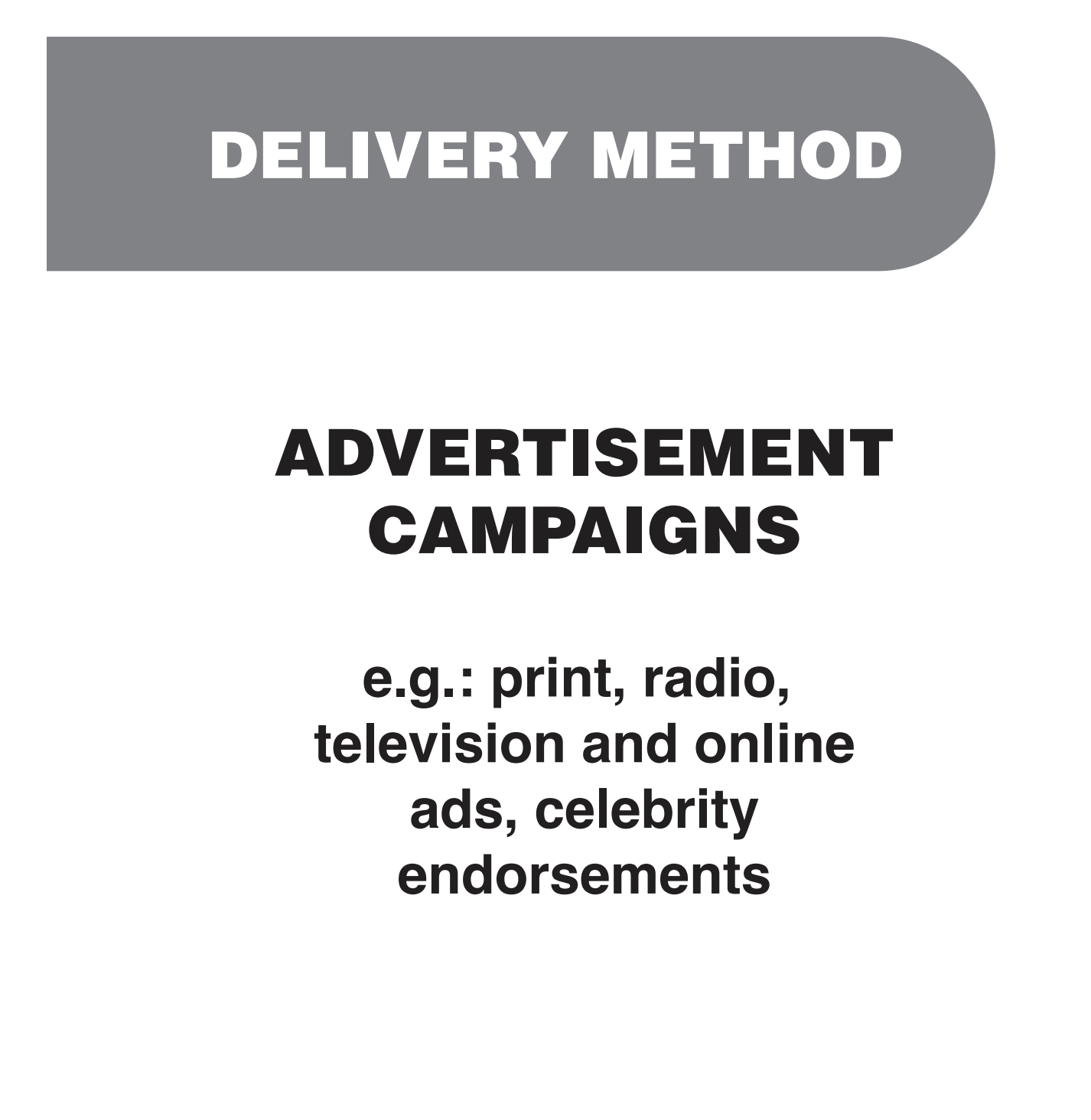


*Supplementary Figure 8: Example of a delivery mode card used in the workshop.*

**Capacity Building Matrix**

The capacity building matrix enabled discussion and ideation on more practical aspects of how challenges could be supported through capacity building ideas. The level of detail achieved in these discussions was facilitated by the structure of the activity and support cards. The matrix is a beneficial and helpful tool to identify and develop the capacity that exactly needs to be developed (Matachi, 2006). The matrix provided the process of developing support at the individual and organizational levels under five main criteria, including: Supports for whom? To do what? and How to develop?, Sustain?, and How to deliver that support?


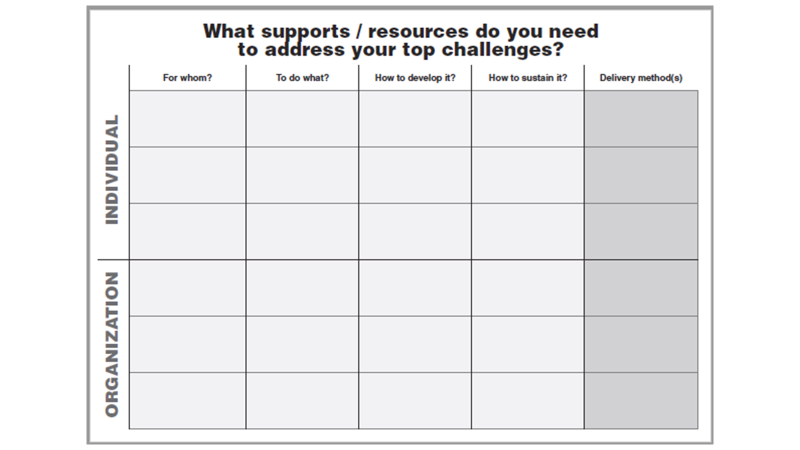


*Supplementary Figure 9: Capacity building matric tool..*
