# Supplementary material for: From design to action: participatory approach to capacity building needs for local overdose response plans
Source: BMC Public Health. 2023 Apr 27;23:774. doi: 10.1186/s12889-023-15414-3 (PMC10132919; doi:10.1186/s12889-023-15414-3)
Supplement: Supplementary file 2 — Additional file 2: Appendix B [file 12889_2023_15414_MOESM2_ESM.docx]

**Appendix B**

**Identified Top priority Support Areas (Challenges)**

***Scenario A***

|  | Scenario A: Plan Development  Plan Development with Lived Experience and Provider Engagement |
| --- | --- |
| Identified Challenges  (Situational Assessment Result) | 1. Access to people with lived/living experiences 2. Different Layers of criminalization and stigma when community partners come together 3. Power dynamic in the meeting (being equitable in the meeting) 4. Policy around paying people with lived/living experiences as an active partner 5. Have a champion at different sectors (e.g., pharmacist, primary care providers) 6. Building relationship, and the ability to maintain relationship (e.g., trust-based collaborative relationships, and addressing change management) 7. Having diverse partners at the table (e.g., clinical and public health sectors) 8. Trust-based collaborative relationships and consensus building techniques |
|  |  |
| Selected challenges  (Workshop participants) | 1. Access to people with lived/living experiences 2. Different Layers of criminalization and stigma when community partners come together 3. Power dynamic in the meeting (being equitable in the meeting) 4. Policy around paying people with lived/living experiences as an active partner 5. Building relationship, and the ability to maintain relationship (e.g., trust-based collaborative relationships, and addressing change management) 6. Having diverse partners at the table (e.g., clinical and public health sectors) |
| Added challenges  (Workshop Participants) | 1. Resources (adequate resources for engagement & programming) (Blank card) 2. Coordination and communication (Blank card) 3. Strategic planning (e.g., Scope, logic model, workplan) (from scenario B) 4. Impacting service accessibility (from scenario B) 5. Tailoring strategies to local context and populations (from scenario B) |
| Prioritized challenges for developing supports | 1. Power dynamic in the meeting (being equitable in the meeting) 2. Different layers of criminalization and stigma when community partners come together 3. Building relationships and the ability to maintain relationship (e.g., trust –based collaboration relationships) 4. Trust-based collaborative relationships and consensus building techniques 5. Tailoring strategies to local context and populations (from scenario B) |

*Supplementary Table 1: Scenario A Identified Top priority Support Areas*

***Scenario B***

|  | **Scenario B: Plan Implementation**  **Plan Implementation with Community Partners in the Local Context** |
| --- | --- |
| Identified Challenges  (Situational Assessment Result) | 1. Strategic planning (e.g., scope, logic model, workplan) 2. Tailoring strategies to local contexts & population (e.g., cultural safety, geographical consideration, cultural and language consideration) 3. Adapting to changing structures, needs, and trends 4. Prioritizing needs and partnership engagement 5. Building relationship, and the ability to maintain relationship 6. Leadership (e.g., senior leadership support, executive leadership meeting with various organizations, and leadership development) 7. Having diverse partners at the table (e.g., clinical sectors & public health sectors) 8. Stigma (e.g., at the individual, coalition, and community levels) 9. Timely access to accurate data & information to communicate data & information 10. Prioritizing data & information (e.g., for urgent action, and access to grassroot data) 11. Capacity of backbone organization (e.g., staffing and facilitation perspectives) 12. Service accessibility (e.g., hours of operation and transportation issue) 13. Tailored evidence-informed practices (e.g., cultural safety, harm reduction, trauma-informed care practices) 14. Knowledge development at the community and professional levels (e.g., ant stigma workshop, training programs) 15. Community access to social skills |
|  |  |
| Selected challenges  (Workshop participants) | 1. Strategic planning (e.g., scope, logic model, workplan) 2. Adapting to changing structures, needs, and trends 3. Leadership (e.g., senior leadership support, executive leadership meeting with various organizations, and leadership development) 4. Having diverse partners at the table (e.g., clinical sectors & public health sectors) 5. Stigma (e.g., at the individual, coalition, and community levels) 6. Timely access to accurate data & information to communicate data & information 7. Prioritizing data & information (e.g., for urgent action, and access to grassroot data) 8. Capacity of backbone organization (e.g., staffing and facilitation perspectives) 9. Service accessibility (e.g., hours of operation and transportation issue) 10. Tailored evidence-informed practices (e.g., cultural safety, harm reduction, trauma-informed care practices) 11. Knowledge development at the community and professional levels (e.g., ant stigma workshop, training programs) |
| Added challenges  (Workshop Participants) | 1. Coordination & communication (Blank card) 2. We don’t have a good discourse on drugs in public spaces (Blank card) 3. Collective action among partners and showing action as well as planning (Blank card) 4. Have a champion at different sectors (from scenario A) 5. Most people who use drugs, use it in a way that is not harmful. Yet the conversation is always only in the harm (Blank card) 6. Lack of empathy for adult with lived experience (Blank card) 7. Around evidence, that is sometimes subjective (Blank card) |
| Prioritized challenges for developing supports | 1. Knowledge development at the community and professional levels/ Prioritizing data & information / Tailored evidence-informed practices 2. Service accessibility (e.g., hours of operation and transportation issue)/ lack of empathy for with lived experience (Blank card)/ Stigma 3. Leadership/ Capacity of backbone organization 4. Having diverse partners at the table/ Having a champion (from scenario A)/ Coordination and communication (Blank card) |

*Supplementary Table 2: Scenario B Identified Top priority Support Areas*

***Scenario C***

|  | **Scenario C: Plan Adaptation**  **Plan Adaptation for Geographic and Cultural Factors** |
| --- | --- |
| Identified Challenges  (Situational Assessment Result) | 1. Tailoring strategies to local context & populations (e.g., cultural safety, geographical consideration, cultural and language consideration) 2. Adapting to changing structures, needs, & trends 3. Severity of the problem and the demographic of the population 4. Dedicated permanent funding 5. Coordination of services and needs-based planning (e.g., Ongoing coordination in capacity to work partners through a facilitation process and identify what’s needed in communities) 6. Strategic planning (e.g., scope, logic model, workplan) 7. Sharing Indigenous perspectives on drug strategy 8. Ongoing access to best practice, and leaders in indigenous approaches 9. There are fewer Indigenous lead organizations in the community 10. Implementing Indigenous pathways 11. Indigenous culturally safe approaches |
|  |  |
| Selected challenges  (Workshop participants) | 1. Tailoring strategies to local context & populations (e.g., cultural safety, geographical consideration, cultural and language consideration) 2. Adapting to changing structures, needs, & trends 3. Severity of the problem and the demographic of the population 4. Coordination of services and needs-based planning 5. Ongoing access to best practice, and leaders in indigenous approaches |
| Added challenges  (By workshop Participants) | 1. People using alone in their home (Blank card) 2. Creating space that people who use drugs are supported to self-organize. so, it should not be that we are creating networks and inviting people with lived/living experience to join. it should be the other way around (Blank card) 3. Supportive housing that doesn't allow people to use in their own home! (Blank card) 4. We ask people to keep doing the work while we are losing people, we can't talk about upstream higher-level process when we have dead people on the street (Blank card) 5. Emergency needs and coordination long term planning (now to not have to compete) (Blank card) 6. Folks don't want their current struggle to affect their future, so they don't want to share their stories (stigma is huge) b- why don't we have a hotline setup so folks can talk about the bad patch they used or the group of friends that overdosed. (Blank card) 7. We don't have a system that allows moderate use (Blank card) 8. Organizations that have service restrictions because they find out people start using again!? (Blank card) 9. Policy around paying people with lived/living expertise (from scenario A) 10. Power dynamic at the meetings (from scenario A) 11. Trust-based collaborative relationship and consensus building techniques (from scenario A) 12. Data for accessing programs and adjusting plans (Blank card) 13. Complementing data with front line narrative to impact planning (Blank card) 14. Developing strategies for when dedication permanent funding is not available (Blank card) |
| Prioritized challenges for developing supports | 1. Coordination of services and need-based planning 2. Ongoing access to best practice, and leads in Indigenous approaches 3. Severity of the problem and the demographic of the population 4. Tailoring strategies to local context & populations (geographical locations) 5. Adapting to changing structures, needs, & trends 6. Data for accessing programs and adjusting plans (Blank card) 7. Complementing data with front line narrative to impact planning (Blank card) 8. Engagement and coordination for long-term planning (Blank card) 9. Developing strategies for when dedication permanent funding is not available (Blank card) |

*Supplementary Table 3: Scenario C Identified Top priority Support Areas*

***Scenario D***

|  | **Scenario D: Plan Sustainability & Iteration** |
| --- | --- |
| Identified Challenges  (Situational Assessment Result) | 1. Knowledge development at the community and professional levels (e.g., anti-stigma workshop, training programs) 2. Access and incorporate new approaches (e.g., out of box practices) 3. Sustainable funding (e.g., having different funding structures) 4. Leadership (e.g., senior leadership support, executive leadership meeting with various organizations, leadership development) 5. Building relationship, and the ability to maintain relationship (e.g., trust-based collaborative relationships, addressing change management) 6. Ongoing communication needs and engagement |
|  |  |
| Selected challenges  (Workshop participants) | 1. Knowledge development at the community and professional levels (e.g., anti-stigma workshop, training programs) 2. Access and incorporate of new approaches (e.g., out of box practices) 3. Sustainable funding (e.g., having different funding structures) 4. Leadership (e.g., senior leadership support, executive leadership meeting with various organizations, leadership development) 5. Building relationship, and the ability to maintain relationship (e.g., trust-based collaborative relationships, addressing change management) 6. Ongoing communication needs and engagement |
| Added challenges  (By workshop Participants) | 1. Access to evidence and practices that looking for sustainability (Blank card) |
| Prioritized challenges for developing supports | 1. Knowledge development at the community and professional levels (e.g., anti-stigma workshop, training programs) 2. Access and incorporate of new approaches (e.g., out of box practices) 3. Access to evidence and practices that looking for sustainability (Blank card) 4. Sustainable funding (e.g., having different funding structures) 5. Leadership (e.g., senior leadership support, executive leadership meeting with various organizations, leadership development) 6. Building relationship, and the ability to maintain relationship (e.g., trust-based collaborative relationships, addressing change management) 7. Ongoing communication needs and engagement |

*Supplementary Table 4: Scenario D Identified Top priority Support Areas*
