# Supplementary material for: From design to action: participatory approach to capacity building needs for local overdose response plans
Source: BMC Public Health. 2023 Apr 27;23:774. doi: 10.1186/s12889-023-15414-3 (PMC10132919; doi:10.1186/s12889-023-15414-3)
Supplement: Supplementary file 3 — Additional file 3: Appendix C [file 12889_2023_15414_MOESM3_ESM.docx]

**Appendix C**

**Capacity Building Matrix: Identified Priority Supports**

*Tables are color coded based on the following project themes:*

| Partnership, Engagement, & Collaborations | Implementation Factors | Data and Information | Evidence and Practice |
| --- | --- | --- | --- |

|  | | | | |
| --- | --- | --- | --- | --- |
| For whom? | To do what? | How to develop it? | How to sustain it? | Delivery methods |
| - Multiple stakeholders:   Lived/living expertise, social service providers, Indigenous groups, community members, researchers and evaluators | - Differentiate between different perspectives - Engage people with their own experiences - Spread the message and influence decisions | - Finding community champion - Developing tools and skills to learn how to facilitate the conversations - Toolkit that include gold standards and best practices | - Open communication channels - Consensus building techniques - Sustainable funding and available resources | - Creating/using social media platforms, advertisement campaigns - Town halls - Community forums |

*Supplementary Table 5: Building relationships and the ability to maintain relationships (e.g., trust-based collaborative relationships)*

|  | | | | |
| --- | --- | --- | --- | --- |
| For whom? | To do what? | How to develop? | How to sustain it? | Delivery methods |
| - At the Ministry of Health and leadership levels | - Change some policies to ensure equity trauma-informed policies | - Keep power dynamic front and center - Shift organizational culture to hire those who represent the people they serve - Enhance anti-racism, anti-oppression, and equity | - Create space for cross- organizational learning - Be responsive to real challenging and changing reality of people who use | - Providing training for both frontline and organizational leaderships |

*Supplementary Table 6: Access to people with lived and living experience*

|  | | | | |
| --- | --- | --- | --- | --- |
| For whom? | To do what? | How to develop it? | How to sustain it? | Delivery methods |
| - People with lived/living expertise - Healthcare workers - Harm reduction workers - Regional representations (EMS, Police, and fire department…) | - Raise awareness - Understand who is at the table and what will be discussed (Topic of conversations) - Ensure mutual respect transparency, disclosure, and equity | - Group guideline in writing (using meaningful terms to ensure a safe place and something to refer) - Using conversational language that is digestible | - Following the terms of agreement - Provide understanding what framework, you are working on - Regularly review and update the agreement | - Group guidelines - Visioning statements |

*Supplementary Table 7: Power dynamics in the meetings (e.g., being equitable in meetings)*

|  | | | | |
| --- | --- | --- | --- | --- |
| For whom? | To do what? | How to develop it? | How to sustain it? | Delivery methods |
| - Local context (diverse people: age and gender) - PWUD and family members - Community members (clinicians, frontline workers, and first responders) | - Influence grass root groups - Meaningful and trust-based engagement - Ongoing evaluation and change (what were wrong and how to improve that) | - Asking peer workers to do outreach and recruitment for committees - Action plan reviewed and updated - Voting mechanism for decision-making structures | - Leadership support and buy-in - Agency endorsement - Mutual respect - Open to new partner and having regular meetings and report back | - Practice reference documents on engaging people with lived experience - Virtual communities - Open space for sharing not government regulated - Training and mentorship |

*Supplementary Table 8: Having diverse partners at the table*

|  | | | | |
| --- | --- | --- | --- | --- |
| For whom? | To do what? | How to develop it? | How to sustain it? | Delivery methods |
| - Leaders in Indigenous communities - Decision-makers - Those who developing programs and services | - Adapt best practices for the community - Engage and get buy-in by community members - Establish credibility | - Involve the leaders & community members - Utilize academic expertise that link to practice “value-based knowledge” - Leveraging Indigenous health leaders | - Funding (sustainable, long-term, and flexible funding) - Monitoring implementation and adaptation as needed - Data to substantiate need | - Mentorship - Print messaging - Practice reference document |

*Supplementary Table 9: Ongoing access to best practices and leaders in Indigenous approaches*

|  | | | | |
| --- | --- | --- | --- | --- |
|  | | | | |
| For whom? | To do what? | How to develop it? | How to sustain it? | Delivery methods |
| - At different levels of the community, including:   Individual, general public, and organizations | - Build knowledge and help address gaps in knowledge & understanding - Reduce stigma - Make change in attitudes (the issue is a health and social issue, not individual issue) | - Building relationship and working with frontline and harm reduction workers - Evidence based practices - Presentations and community forums | - Continuing information sharing - Training - Evaluation tools (e.g., measuring attitudes and values check-ins) | - Workshops - Training modules (e.g., Train the Trainers) - Resource documents - Expanding existing programs & evidence dissemination |

*Supplementary Table 10: Knowledge development at the community and professional levels*

|  | | | | |
| --- | --- | --- | --- | --- |
| For whom? | To do what? | How to develop it? | How to sustain it? | Delivery methods |
| - PWUD - Community members - Frontline workers & first responders - Public health practitioners - Policy makers & Funders - Researchers | - To find accurate and reliable data promptly (provide data that reflect on the ground experiences) - Build relationships that influence system & policy change - Providing alert system that gets to all who need the information | - Contextualize data so everyone involved can understand it (people with lived experience) - Reading data with people with lived/living experiences to make sure they agree with findings - Recognize value of different resources | - Building paying people into the budget | - Alert system (can help to collect data for both the individuals and organizations) |

*Supplementary Table 11: Prioritizing data & information (e.g., for urgent action and access to grassroot data)*

|  | | | | |
| --- | --- | --- | --- | --- |
| For whom? | To do what? | How to develop it? | How to sustain it? | Delivery methods |
| - PWUD and family & friends who support them - Community - Frontline workers | - Remove restrictions to access - Reduce gaps in the system (e.g., transportation support, mobile outreach, and private homes) - Add more service locations) | - Physicians engagement (referral system) - Partnering with community agencies (upstream) - Funding increase (from all level of the government) | - Relationship building (trust-based) - Inclusive service provider training (anti-stigma, anti- discrimination, and cultural-safety) - Collaboration and work with different settings (e.g., hospitals, housing, and long-term care) | - 24/7/365 access to community hub - Experiential workers on site |

*Supplementary Table 12: Service Accessibility (e.g., hours of operation and translation issues)*

| Table 9 Coordination of services and needs-based planning | | | | |
| --- | --- | --- | --- | --- |
| For whom? | To do what? | How to develop it? | How to sustain it? | Delivery methods |
| - Youth and adult | - Easy access to services - Reduce barriers by creating services based on geographic areas | - Having adaptable and flexible plans (based on geographic areas and communities’ needs) - Ensure community involvement and input | - Funding (sustainable, long-term, and flexible funding) - Ongoing dialogues and accountability - Sharing of what is happening across the province | - Recruitment training programs - Virtual community |

*Supplementary Table 13: Coordination of services and needs-based planning*

| Table 10 Developing strategies for when dedicated and permanent funding is not available | | | | |
| --- | --- | --- | --- | --- |
| For whom? | To do what? | How to develop it? | How to sustain it? | Delivery methods |
| - All staff | - Resources sharing that can build the capacity of all involved staff - Advocacy for funding and policy shifts - Guidance on best practices | - Creative spending/sharing for resources - Sharing and collaboration | - N/A | - Face to face - Mentorship - Webinars |

*Supplementary Table 14: Developing strategies for when dedicated and permanent funding is not available*

|  | | | | |
| --- | --- | --- | --- | --- |
| For whom? | To do what? | How to develop it? | How to sustain it? | Delivery methods |
| - All involved stakeholders | - Create sustainability for people around the table | - By better alert system - Create & use social media platforms and online forums - Increasing visibility (e.g., advertisement, and Champaign) | - Having good facilitators, communicators, and lived/living expertise - Adaptability and open minded | - N/A |

*Supplementary Table 15*: *Ongoing communication needs and engagement*

|  | | | | |
| --- | --- | --- | --- | --- |
| For whom? | To do what? | How to develop it? | How to sustain it? | Delivery methods |
| - Among PWUD, frontline workers, people in community (employees & employers),   And anyone who has interest to be part of the plan | - Community improvement and more initiatives - Make sure leadership is changing and evolving | - Mentorship and having practice support documents - Consistent communication for alerts and reporting system for the streets (Apps for alert) - Internal structure that take on training | - Outreach - Funding - Cost analysis and urgent categories | - Social media - Mentorship - Advertainment campaigns |

*Supplementary Table 16*: *Leadership (senior leadership support, executive leadership meeting with various organizations and leadership development)*
